# Deficient or R273H and R248W Mutations of p53 Promote Chemoresistance to 5-FU *via* TCF21/CD44 Axis-Mediated Enhanced Stemness in Colorectal Carcinoma

**DOI:** 10.3389/fcell.2021.788331

**Published:** 2022-01-05

**Authors:** Xiaolei Gao, Xuan Zheng, Yixin Zhang, Liying Dong, Liangjie Sun, Na Zhao, Chong Ding, Zeyun Ma, Yixiang Wang

**Affiliations:** ^1^ Central Laboratory, Beijing, China; ^2^ Department of Oral and Maxillofacial Surgery, Beijing, China; ^3^ National Engineering Laboratory for Digital and Material Technology of Stomatology, Beijing, China; ^4^ Beijing Key Laboratory of Digital Stomatology, Beijing, China; ^5^ Department of Restorative Dentistry and Biomaterials Sciences, Harvard School of Dental Medicine, Boston, MA, United States; ^6^ Shanghai Stomatological Hospital, Fudan University, Shanghai, China; ^7^ Department of VIP Service, Peking University School and Hospital of Stomatology, Beijing, China

**Keywords:** colorectal carcinoma, deficient or mutant p53, R273H, R248W, stemness, TCF21/CD44 axis, chemoresistance

## Abstract

**Background:** p53 mutations are highly frequent in various human cancers and are reported to contribute to tumor malignance and chemoresistance. In this study, we explored the mechanism by which mutant p53 promotes carcinogenesis and chemoresistance and provided novel insights into cancer therapy.

**Materials and methods:** A total of 409 patients with colorectal carcinoma from TCGA database were subdivided into two groups according to the p53 status, namely, mutant p53 and wild-type p53, following with GSEA analysis. The differences of the clinicopathologic index were also analyzed. Two HCT116 cell lines containing hot spots at codons R273H and R248W of p53 were constructed based on HCT116 with knockout p53, respectively. Cell viability, mobility, clonogenesis, and stemness were detected by CCK8, transwell migration and invasion, colonogenic, and sphere formation assays. Resistance to 5-FU was examined by live-dead staining and flow cytometry. qPCR, Western blot, and luciferase reporter assay were performed to identify that deficient or mutant p53 promoted chemoresistance of the colorectal carcinoma cell line HCT116 through the TCF21/CD44 signaling pathway, with the following rescue assays by overexpression of TCF21 and knockdown of CD44.

**Results:** Patients with recurrence harbor a higher frequency of mutant p53 than those without recurrence (*p* < 0.05). The mutant p53 group developed a larger tumor than the wild-type one. GSEA analysis showed that oncogenic signatures were enriched in the mutant p53 group. Extracellular assays showed that cancer cells with deficient or mutant p53 (R273H and R248W, respectively) promoted colon cancer cell growth, migration, invasion, and stemness. The mutant cancer cells were also observed to be significantly resistant to 5-FU. Xenografts also confirmed that HCT116 cells harboring deficient or mutant p53 promoted cancer growth and 5-FU tolerance. Luciferase reporter assay showed that deficient or mutant p53 R237H and R248W endowed cancer cells with chemoresistance by activating CD44 *via* repressing the nuclear transcription factor TCF21 expression. Overexpression of TCF21 or knockdown of CD44 could rescue the sensitivity to 5-FU in deficient and mutant p53 HCT116 cell lines.

**Conclusion:** Our results, for the first time, reveal a novel deficient or mutant p53/TCF21/CD44 signaling pathway which promotes chemoresistance in colorectal carcinoma. The axis could be an effective therapeutic strategy against deficient- or mutant p53-driven chemoresistance.

## Introduction

p53 exerts a key role in regulating cell fate by orchestrating genes with functions in apoptosis, cell cycle, senescence, differentiation, survival, and DNA damage responses (Riley et al., 2008). The International Agency for Research on Cancer (IARC) p53 database indicates that only 7% of all mutants in human cancers are fully functional (http://www-p53.iarc.fr/). Missense mutations generated by single nucleotide changes are the most common manner of inactivating p53. A myriad of evidence suggest a powerful connection between the p53 activity and mutational status, which demonstrates that the inactivated mutations in p53 significantly contribute to carcinogenesis and malignant transformation. These data indicate the need for deciphering the implication of mutant p53 in tumor development and chemoresistance of individual substitutions, especially fully functional mutations. On the one hand, mice with the homozygous mutant of p53 R273H and R248W also manifest an increased risk for metastatic cancers, accompanying with the ubiquitous disruption of the DNA damage sensor ataxia-telangiectasia–mutated (ATM) signaling (Song et al., 2007; Liu et al., 2010). On the other hand, several molecules and signaling pathways were reported in mutant p53-mediated chemoresistance, such as activation of NF-κB, MGMT, SLC25A1, PELP1, EFNB2, and ErbB2, and so on (Scian et al., 2005; Kolukula et al., 2014; Wang et al., 2014; Krishnan et al., 2015; Alam et al., 2016). Although significant pro-tumor and -chemoresistant impacts of mutant p53 have been studied in various tumors, the underlying mechanisms were still far from understandable.

Cancer stem cells (CSCs) are strongly involved in chemoresistance, which is the major culprit for the poor outcome in colorectal cancer (CRC) (Skarsson et al., 2014; Van der Jeught et al., 2018). In recent decades, mutant p53 has been reported to have gained a novel function in inducing dedifferentiation of somatic cells to CSCs, raising considerable postulation if mutant p53 enhances chemoresistance through mediating cancer cell stemness. The common gene signature and transcription factor shared by embryonic stem cells and breast undifferentiated tumors also strengthened this notion. Many studies have suggested that inactivation of p53 leads to cancer stemness. Pinho et al. observed stemness features including elevated sphere formation and expression of CSC makers in pancreatic cells with homozygous p53 deletion (Pinho et al., 2011). Loss of p53 could trigger cellular plasticity from somatic cells to pluripotent cells through activating nestin in liver cancer (Tschaharganeh et al., 2014). However, only a few pieces of evidence clearly described the role of mutant p53 in driving the CSC phenotype. The possible novel plastic property of mutant p53 in cancer cell stemness was firstly proposed by Sarig’s research (Sarig et al., 2010). Grespi et al. further identified a cluster of mutant p53-dependent mRNAs that participated in the transition from embryonic fibroblasts to induce pluripotent stem cells (Grespi et al., 2016). A few molecules were proposed to participate in mutant p53-driven cancer stemness, such as PI3K/AKT2 and YAP/TAZ (Escoll et al., 2017).

In this study, we focused on the two hot spot alleles R273H and R248W, which show two of the highest mutant frequency than other codons in common human cancers (more than 6%, IARC TP53 Database). We constructed R273H and R248W homozygous mutants of p53 based on the p53-null HCT116 cell line to investigate the underlying mechanism regarding p53-mediated chemoresistance. First and foremost, our results proposed that CD44 was strikingly implicated in mutant p53-induced cancer stemness, which could be a promising target for colorectal cancer treatment. Second, we, for the first time, identified that deficient or mutants of p53 R273H and R248W activated CD44 by inactivating TCF21, which encodes a transcription factor of the basic helix–loop–helix family. Finally, we demonstrated that mutant p53 not only conferred the aggressive manifestations of HCT116 cells but also protected HCT116 cells from chemotherapeutic cytotoxicity *via* the p53/TCF21/CD44 signaling pathway.

## Materials and Methods

### Data Acquisition

The original datasets and the corresponding clinicopathological features including age, gender, tumor stage, lymphatic metastasis, and lymphovascular and perineural invasion were downloaded from TCGA database (https://tcga-data.nci.nih.gov/tcga/). Gene mutation information was downloaded from the cBioPortal database (http://www.cbioportal.org/). A total of 409 colorectal carcinoma cases were included for subsequent analysis. We downloaded and analyzed the study data in accordance with the relevant data policies of TCGA database dataset, and therefore, no additional ethics approval was needed.

### Gene Set Enrichment Analysis

GSEA was performed in mutant p53 and wild-type p53 subgroups, by GSEA software with the criteria *p* < 0.05 and FDR<0.25 (Subramanian et al., 2005) and visualized by clusterProfiler packages of R (Yu et al., 2012).

### Cell Line Generation and Transfection

The human colorectal carcinoma cell line HCT116 p53 (+/+) was purchased from the American Type Culture Collection (ATCC CCL-247). The p53-null HCT116 p53 (-/-) cell line was gifted by Dr. Bert Vogelstein (Johns Hopkins University School of Medicine, Baltimore, MD, United States). The p53-null HCT116 p53 (-/-) cells were transduced HCT116 stable expressing p53 R273H and p53 R248W cell lines and were generated by transduction with pCMV-Neo-Bam mutp53 plasmids expressing mutant p53 with R273H or R248W into HCT116 p53 (-/-) cells (pCMV-Neo-Bam-p53 R273H and R248 mutation vectors were a gift from Dr. Bert Vogelstein (Addgene plasmids #16437 and #16439), G418 selection and single colony screening, respectively. A control stable HCT116 cell line was established by transducing with empty vectors followed by G418 screening. The human colorectal carcinoma cell line SW480 (harbors mutp53 at codon 273, R–H) was gifted by the National Collection of Authenticated Cell Cultures (China).

siRNAs specific against human CD44 and p53 were synthesized by RiboBio Co., Ltd. (Guangzhou). To avoid off-target effects, three different siRNAs against CD44 were employed (stB0002525A-C).

The p53 R273H and p53 R248W HCT116 cell lines were transduced with the TCF21 overexpression plasmid to establish cells with the transient ectopic expression of TCF21 for rescue assay. The TCF21 overexpression plasmid was constructed in the pCMV6-entry vector using the subcloning method in this study. The above transfection assays were performed with Lipo8000^TM^ according to the manufacturer’s instructions (Beyotime, Shanghai, China, and C0533).

### CCK-8 Assay

The CCK-8 kit (Dojindo, Shanghai, China) was used to measure proliferation of HCT116 cell lines. A total of 5,000 cells in a volume of 100 μl per well were cultured in five replications in a 96-well plate. The CCK-8 was mixed with medium (10 μl per well) into each well and incubated at 37°C, 5% CO_2_ for 30 min. The cell proliferation rate was determined by the measurement of the OD_450_ value at 0, 24, 48, 72, and 96 h after cell inoculation.

### Transwell Migration and Invasion Assays

HCT116 cells were starved for 24 h, then trypsinized, counted, and seeded at 2 × 10^4^ cells/200 μl DMEM medium without serum in the upper chambers of transwell with and without diluted Matrigel-coated membranes for invasion and migration, respectively (8.0 μm pore, Millipore, Bedford, MA, United States; 25 μg/ml Matrigel, BD, Minneapolis, MN, United States). For the assay, 500 μl DMEM medium containing 10% fetal bovine serum (FBS) was added to the bottom chambers. At 30 h post-inoculation, the cells were washed by phosphate-buffered saline (PBS) three times and fixed by ethanol for 20 min. Then, the cells were stained with 0.1% crystal violet for 30 min at room temperature. After removing the cells on the membrane of the upper chamber, the invaded cells were counted using an orthographic microscope at ×20 magnification (Olympus, Tokyo, Japan).

### Colony Formation Assay

Each cell line was plated in a 24-well plate at 200 cells/well and cultured for 2 weeks. The colonies were washed with PBS three times, fixed with ethanol, and stained with 0.1% crystal violet. The colony number was counted using an inverted microscope at ×40 magnification (Olympus, Tokyo, Japan).

### Tumorsphere Formation Assay

HCT1116 cells were trypsinized to generate a single cell suspension and plated to a final volume of 500 μl of serum-free medium [DMEM/F12 [1:1] with B27 (Invitrogen, Carlsbad, CA, United States), 20 ng/ml bFGF (Invitrogen), 20 ng/ml EGF (Invitrogen)] in one well of a 24-well low attachment plate, and with 1,000 cells per well. The tumorspheres were cultured for 10 days and counted at ×40 magnification (Olympus, Tokyo, Japan).

### Reverse Transcription and qPCR Analysis

Total RNA from cells was extracted using the TRIzol reagent (Invitrogen) following the manufacturer’s protocol. RNAs were reverse-transcribed into cDNAs by using the Reverse Transcription kit (Promega, Madison, WI, United States). Real-time quantitative PCR (qPCR) was performed using the SYBR Green master mix (Roche Diagnostics, Indianapolis, IN, United States) with the qPCR instrument (Bio-Rad, Hercules, CA, United States). The gene-specific primer sets were used at a final concentration of 0.5 µM. All real-time qPCR assays were performed in triplicates and repeated three times. Relative expression values of each target gene were normalized to the RPS18 mRNA level. Sequences used were as follows:

RPS18 (forward: 5′-GCG​GAA​AAT​AGC​CTT​TGC​CAT-3′; reverse: 5′-TGA​TCA​CAC​GTT​CCA​CCT​CAT-3′), TYMS (forward: 5′-GGA​GTG​AAA​ATC​TGG​GAT​GCC-3′; reverse: 5′-ACT​GGA​AGC​CAT​AAA​CTG​GGC-3′), TCF21 (forward: 5′-TCC​TGG​CTA​ACG​ACA​AAT​ACG​A-3′; reverse: 5′-TTT​CCC​GGC​CAC​CAT​AAA​GG-3′), DPYD (forward: 5′-GGC​GGA​CAT​CGA​GAG​TAT​CCT-3′; reverse: 5′-TTC​TTG​GCC​GAA​GTG​GAA​CAC-3′), UPP1 (forward: 5′-CAC​TTG​CCC​CGT​CAG​ACT​TTT-3′; reverse: 5′-CAC​CAA​CGC​ACC​TGA​TGA​AG-3′), TYMP (forward: 5′-GGT​GTG​GGT​GAC​AAG​GTC​AG-3′; reverse: 5′-GCA​GCA​CTT​GCA​TCT​GCT​C-3′), CD44 (forward: 5′-AAC​CTG​GGA​TTG​GTT​TTC​ATG​G-3′; reverse: 5′-TGC​CAT​TTC​TGT​CTA​CAT​CAG​T-3′), LGR5 (forward: 5′-AAC​ATC​AGT​CAG​CTG​CTC​CC-3′; reverse: 5′-GTT​AGC​ATC​CAG​ACG​CAG​GG-3′), EPCAM (forward: 5′-GTC​ATT​TGC​TCA​AAG​CTG​GC-3′; reverse: 5′-GCT​CTC​ATC​GCA​GTC​AGG​AT-3′), LIN28A (forward: 5′-TCA​AAA​GGA​GAC​AGG​TGC​TAC-3′; reverse: 5′-AAA​AGA​ATA​GCC​CCC​ACC​CAT​T-3′), OCT4 (forward: 5′-TGG​GGG​TGA​TAC​TTG​AGT​GAG-3′; reverse: 5′-TGG​CTG​AAT​ACC​TTC​CCA​AAT-3′), and SOX2 (forward: 5′-TTT​GTC​GGA​GAC​GGA​GAA​GC-3′; reverse: 5′-TAA​CTG​TCC​ATG​CGC​TGG​TT-3′).

### Western Blot

Cells were washed three times with pre-cooling PBS and then suspended in a cell lysis buffer (Cat No: P0013, Beyotime, Shanghai, China) with protease inhibitors. Protein concentrations were quantified using the Bradford Protein Assay Kit (Beyotime, Shanghai, China) according to the manufacturer’s instructions. For the assay, 40 μg of protein per sample was prepared for the following steps, including gel electrophoresis, transferring to polyvinylidene difluoride membrane, blocking for 1 h at 25°C with 5% nonfat milk, binding with antibodies, and detecting with an ECL detection system (Applygen Technologies, Beijing, China).

The following antibodies for the Western blot analysis were used: the anti-p53 antibody (Abcam, United States; ab1101, dilution: 1:5,000); antibody against TYMS (ABclonal, Wuhan, China, A10441, and dilution: 1:2000); anti-TCF21 antibody (ABclonal; A17451, dilution: 1:2000); anti-CD44 antibody (ABclonal; A1351, dilution: 1:2000); anti-cleaved PARP1 (ABclonal; A0942, dilution: 1:1,000); and anti-cleaved caspase 3 (Cell signaling technology, Danvers, MA, United States; #9664S, dilution: 1:1,000). The anti-RPS18 antibody was used for internal control protein (ABclonal; A11687, dilution: 1:1,000).

### Live/Dead Cell Viability Assay

Cells were inoculated into a 24-well plate with 4 × 10^5^ cells per well and treated with 5-FU (100 μM/L per well, Jinyao, Tianjin, China, and H12020959) for 24 h. The cells were washed twice with PBS, and live and dead cells were measured using a LIVE/DEAD® Viability/Cytotoxicity Assay Kit (Invitrogen) according to the manufacturer’s instructions. The results were observed by fluorescence microscopy (Olympus, DP700).

### Flow Cytometry

Each cell line was resuspended in PBS containing 2% FBS and stained with fluorescent-conjugated antibodies against CD44 (5 μl per sample, BioLegend, San Diego, United States, and Cat No: 338803) for 30 min at 4°C. The IgG1 isotype was used as negative control (BioLegend; Cat No: 400107). Then, the samples were analyzed by using a flow cytometer instrument.

### Apoptosis Analysis

The cells were added to a 6-well plate with 5 × 10^5^ cells per well. After 24 h, the cells were treated with 5-FU (100 μmol/L per well) for 24 h. Then, the cells were dissociated and harvested by 0.25% trypsin without EDTA, and apoptosis detection was performed using the Annexin V-FITC/PI Apoptosis Detection Kit (Solarbio, Beijing, China). Briefly, the cells were washed with PBS and resuspended in 100 μl 1 × binding buffer. Then, the cells were incubated with the FITC-conjugated Annexin V antibody (5 μl per sample) for 15 min at room temperature in a dark place. Finally, propidium iodide (PI) staining solution was added to the mix (5 μl per sample) and analyzed by using a flow cytometer instrument immediately.

### Xenograft Tumorigenicity and Chemoresistance Assays

Six-week-old male BALB/c male nude mice (*n* = 40) were purchased from Vital River Laboratory Animal Co., Ltd. (Beijing, China). The animal experiments were approved by The Institutional Animal Care and Use Committee at Peking University Health Center (LA 2019–309). All 40 mice were randomly subdivided into four groups and underwent subcutaneous injection of different viable cell suspension mixture (resuspended with PBS, 5 × 10^6^ per mouse). At 20 days after tumor cell transplantation, the mice were subjected to 5-FU treatment (20 mg/kg of weight) continuously for 10 days. The tumor size was measured using calipers once a week for 4 weeks. Tumor volume = ½ (length × width^2^). All mice were sacrificed on the day after drug withdrawal.

### Immunohistochemistry

The tumors were harvested, formalin-fixed, paraffin-embedded, and made into slides. Then, the slides were deparaffinized in xylene and then were rehydrated through an ethanol gradient. The sections were heated at 100°C in 0.01 mol/L sodium citrate buffer (pH 6.0) for 10 min for antigen retrieval. The slides were inactivated with 3% H_2_O_2_ for 15 min at room temperature in the dark place, blocked with 10% goat serum for 10 min at 37°C, and incubated with 100 µl primary antibodies diluted in PBS to each section overnight at 4°C, 100% humidity. Next day, the sections were stained with horseradish peroxidase (HRP)–labeled secondary antibody (ZSGB-BIO, Beijing, China) at 37°C for 40 min. Finally, the sections were incubated with 3,3′-diaminobenzidine (DAB, ZSGB-BIO, Beijing, China, and ZLI-9019) to perform color development. After counterstaining with hematoxylin, the sections were observed and recorded using a microscope with an Olympus DP controller (Olympus). A staining score was calculated by multiplying two scores of staining intensity (0: negative staining; 1: weak staining; 2: moderate staining; 3: strong staining) and intensity (0: 0–25%; 1: 25–50%; 2: 50–75%; 3: >75%) of positive stained colorectal cancer cells.

### Luciferase Reporter Assay

CD44 promoter regions containing 2 motifs that can bind to TCF21 protein were predicted using JASPAR (http://jaspar.genereg.net/). pGL3-CD44 with a wild-type CD44 promoter (pGL3-CD44wt) and pGL3-CD44 with a CD44 promoter with deleted TCF bind sites (pGL3-CD44mut) were constructed. pGL3-CD44wt, pGL3-CD44mut, or pGL3-Basic were transiently co-transfected with pTK-RL plasmids into different p53 status HCT116 cell lines (5 × 10^5^) plated in a 6-well plate using the Lipo8000^TM^ (Beyotime, Shanghai, China, and C0533) transfection reagent. At 48 h post-transfection, the cells were harvested, and the assay was performed with the Dual Luciferase Assay kit (Promega, Madison, WI, United States) according to the manufacturer’s instructions. Luciferase activities were normalized to the relative ratio of firefly luciferase activity to renilla luciferase activity. All experiments were performed in triplicates and repeated three times.

### Statistical Analysis

Statistical analyses were performed using GraphPad Prism 8.4. *p* < 0.05 was considered statistically significant. A nonparametric *t* test was performed to compare continuous variables, and the χ^2^ test was used to compare categorical variables between two groups. The ANOVA test was utilized to compare more than two groups.

## Results

### Missense Mutations in p53 Are Related With Cancer Recurrence and Malignance in Colorectal Carcinoma Patients

**FIGURE 1:**
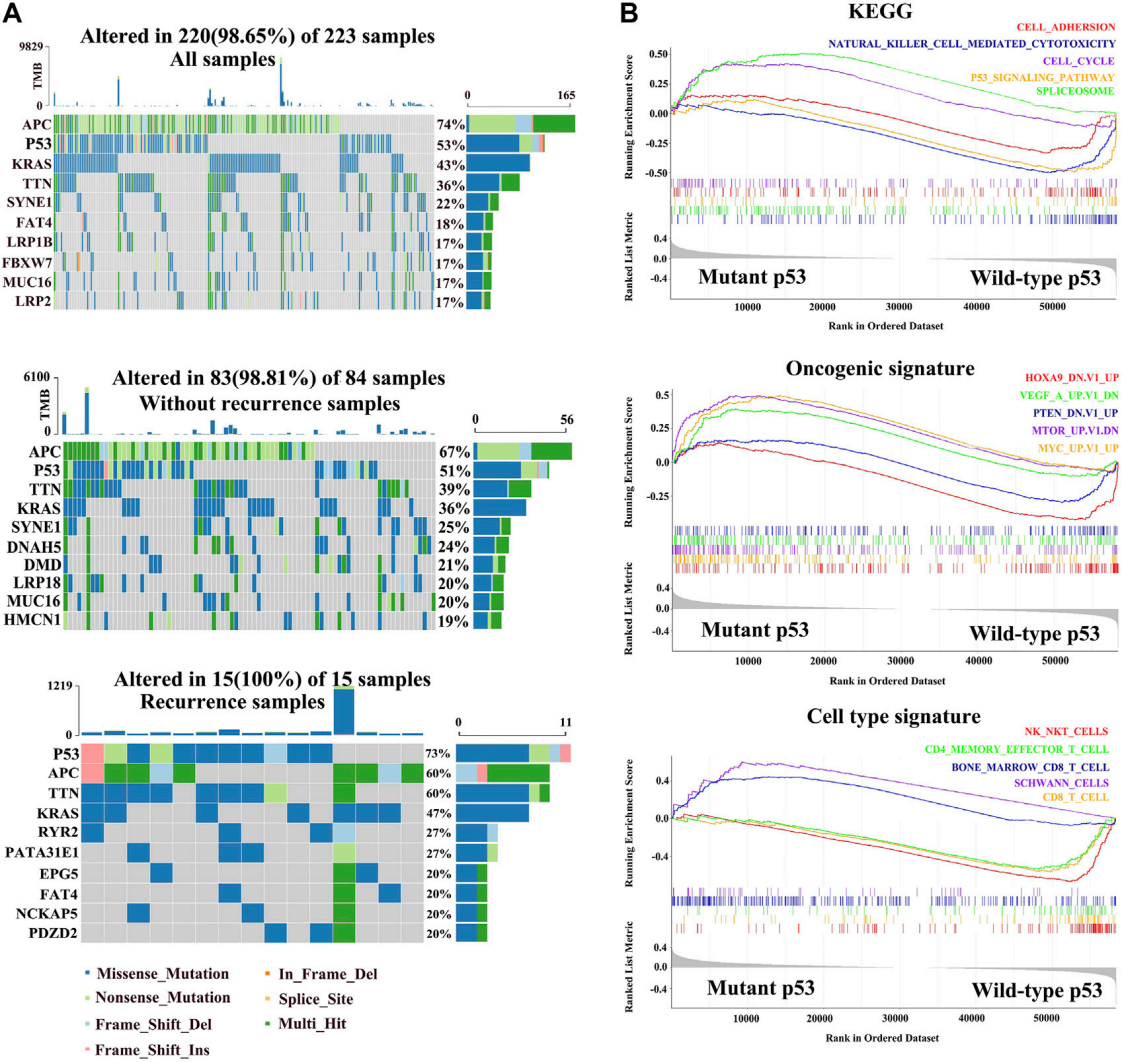
Molecular characteristics of different p53 statuses of colorectal carcinoma in TCGA. **(A)** Genomic mutation signature in the patients with colorectal carcinoma (COAD) from TCGA database. The upper panel shows the mutation percentage of p53 in all COAD samples. The middle panel shows the mutation percentage of p53 in tumor-free samples. The bottom panel shows the mutation percentage of p53 in with-tumor samples. **(B)** Gene set enrichment in mutant p53 and wild-type p53 groups, respectively (*p* < 0.05, FDR < 0.25).

To study the contribution of the p53 status to carcinogenesis, we downloaded colorectal carcinoma (COAD) from TCGA database and analyzed the status of p53 in paired primary tumor samples obtained from 223 COAD patients with and without recurrence, according to the cBioPortal database. The total mutation rate of p53 in all COAD samples is 53%, which is analogous to the tumor samples without recurrence (51%). However, the mutated frequency of p53 rocketed to 73% in COAD recurrent samples (Figure 1A). We next explored the relationship between the p53 status and clinical characteristics and found that patients with mutant p53 showed larger tumors and were prone to lymphatic metastasis (*p* < 0.001, Table 1).

**TABLE 1:** Differences of clinical pathological characteristics between missense p53 and wild-type p53 groups.

	Missense	WT	*p* Value
	*n* = 155	*n* = 254	
Age	66.32 ± 12.89	67.35 ± 13.12	0.439
Gender			
Male	85	134	0.682
Female	70	120	
Tumor stage			0.0003**
I-II	68	163	
III-IV	79	88	
lymphatic metastasis			0.0004**
with	79	84	
without	76	170	
Lymphovascular invasion			>0.99
with	54	89	
without	85	140	
perineural_invasion			0.0745
with	26	16	
without	54	65	

Note: *p < 0.05, **p < 0.01, ***p < 0.001, and ****p < 0.0001.

To investigate the potential mechanism by which mutated p53 contributes to colorectal cancer development, we divided the COAD patients into wild-type p53 and missense p53 subgroups, and GSEA and cell enrichment analysis were performed on them. The KEGG results showed that the cell cycle and spliceosome pathways were enriched in the mutation groups, while adhesion molecules, natural killer cell–mediated cytotoxicity, and p53 signaling pathways were activated in the wild-type group. Oncogenic signature showed that oncogenes including MYC, MTOR, and VEGF were upregulated in the mutant group, while cancer suppressor genes including PTEN and HOXA9 were significantly downregulated in wild-type p53. Importantly, cell type signature showed that Schwann cells and pro-B cells were also increased in the mutated cohort, while natural killer T cells, CD8 T cells, and CD4 effector T cells were activated in the wild-type group (Figure 1B).

### Deficient, R273H, and R248W Missense Mutations of p53 Promote Colorectal Cancer Cell Growth, Mobility, and Stemness.

**FIGURE 2:**
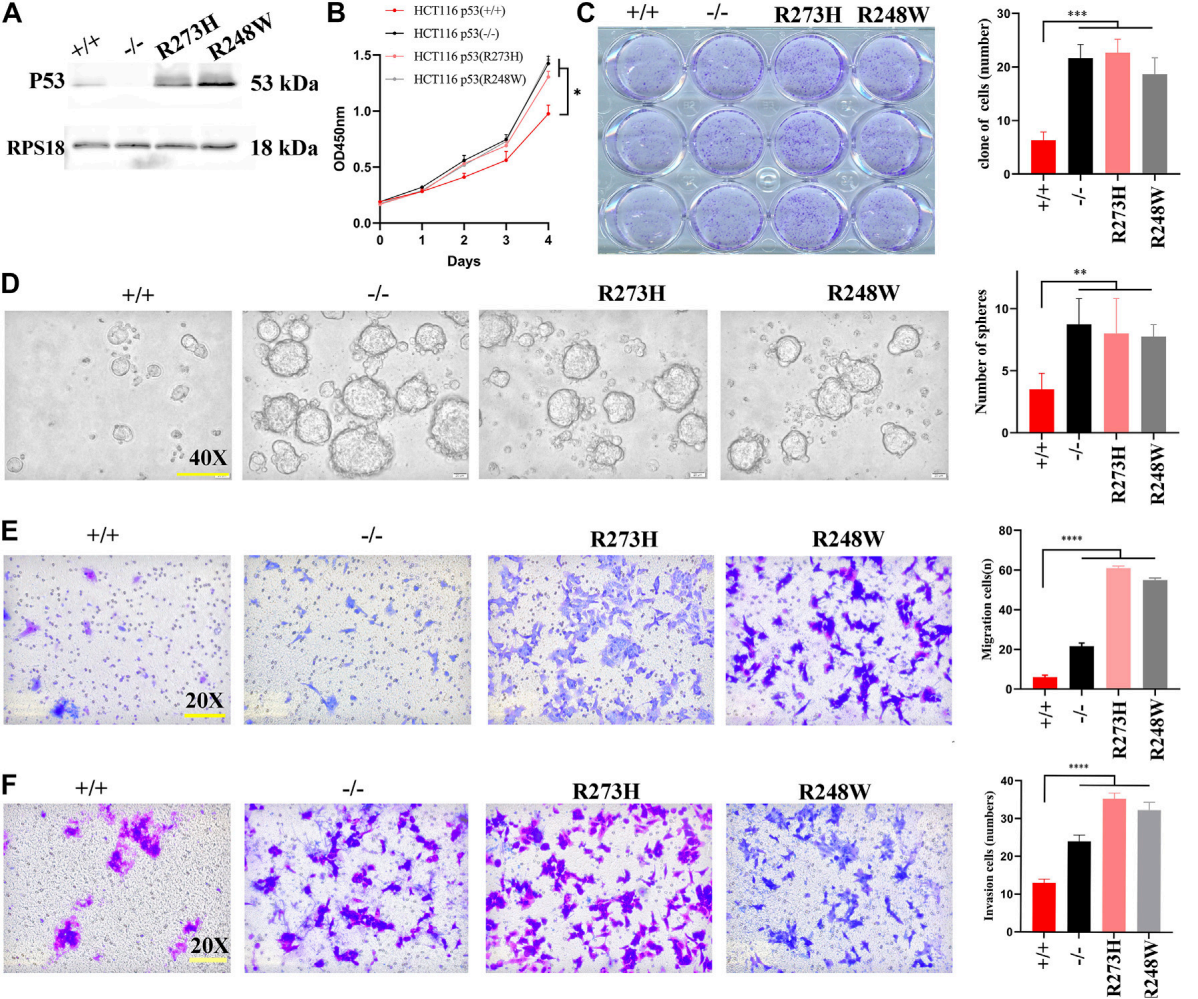
Cell growth, migration, invasion, and stemness in HCT116 with different p53 statuses. **(A)** Images of Western blot for p53 in HCT116 p53 (+/+), p53 (-/-), p53 (R273H), and p53 (R248W). RPS18 was used as internal control. **(B)** Growth rate of the different statuses of p53 in HCT116. **(C)** Images of colonogenic formation in HCT116-derived cell lines with different p53 status. **(D–F)**. Images of tumorsphere formation, migration, and invasion in each HCT116-derived cell lines. *p < 0.05, **p < 0.01, ***p < 0.001, ****, and *p* < 0.0001. The error bar was from three independent experiments.

First, we constructed two HCT116-derived cell lines with mutant “hot spots” of p53, R273H and R248W, based on deficient p53 HCT116 cells (HCT116 p53 (-/-), examined using Western blot (Figure 2A). Next, we performed CCK8, colonogenic, tumorsphere formation, and transwell migration and invasion assays to compare the behaviors among HCT116-derived cancer cell lines with different statuses of p53. CCK8 and colony formation assays showed that HCT116 p53 (-/-), p53 (R273H), and p53 (R248W) had higher proliferation and clonogenic rates than HCT116 p53 (+/+) (*p* < 0.05, Figures 2B,C). Sphere formation assay displayed that increased sphere rates were observed in deficient and mutant p53 HCT116 cells (*p* < 0.01, Figure 2D), suggesting an increased stemness in HCT116 p53 (-/-), p53 (R273H), and p53 (R248W) cells. Transwell migration and invasion assays showed enhanced cell mobilities, including migration and invasion, in HCT116 p53 (-/-), p53 (R273H), and p53 (R248W) (*p* < 0.001, Figures 2E,F). These data suggested that mutant p53 endowed colon cancer cells with more aggressive and malignant phenotypes than wild-type ones.

### R273H and R248W Mutants in p53 Enhance 5-FU Resistance in HCT116

**FIGURE 3:**
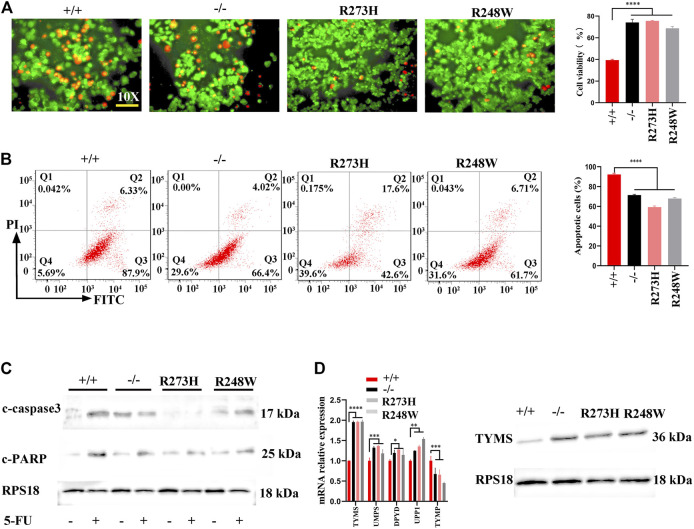
Anti-apoptosis and 5-FU resistance in HCT116 with different p53 statuses. **(A)** Representative images for live-dead staining in HCT116-derived cell lines. Red dot represents dead cells, and green dot shows alive cells. **(B)** Representative images for FITC-Annexin V/PI flow cytometry in HCT116-derived cell lines. The areas of Q2 and Q3 are considered as apoptotic cells. **(C)** The expressions of cleaved-caspase3 and cleaved PARP protein in HCT116-derived cell lines. **(D)** mRNA and protein levels of TYMS in different HCT116-derived cell lines. ****p < 0.0001. The error bar was from three independent experiments.

The elevated stemness within mutant p53 cancer cells allowed us to investigate whether the changes were involved in colon cancer chemoresistance; we detected the apoptosis of HCT116 cells with different p53 statuses after 5-fluorouracil (5-FU) treatment. Live-dead staining showed increased cell viability both in knockout and mutated p53 cells (*p* < 0.0001, Figure 3A). Flow cytometry further demonstrated significant increased anti-apoptosis cells in the two mutated cancer cells (*p* < 0.0001, Figure 3B). Another human colorectal carcinoma cell line SW480 also exhibited the same anti-apoptosis to 5-FU treatment (*p* < 0.0001, Supplementary Figures S1A,B). CCK8 results also verified the attenuated killing effect of 5-FU in HCT116 p53 (-/-), p53 (R273H), and p53 (R248W) (Supplementary Figure S2). Western blot further examined cleaved-PARP and cleaved-caspase3 expression levels; the two key factors involved in cell apoptosis showed a decreased level in knockout and mutated p53 HCT116 cells (Figure 3C). Cleaved-caspase 3 was also downregulated in mutant p53 SW480 cell lines (Supplementary Figure S1C). qPCR and Western blot were then used to check thymidylate synthase (TYMS), the direct target of 5-FU, and affected the sensitivity of colon cancer cells to 5-FU. As expected, TYMS was overexpressed in p53 mutant HCT116 and SW480 cell lines, both at mRNA and protein levels (Figures 3D,E; Supplementary Figure S1C). Together, these data suggested that loss-of-function and mutated p53 both could enhance cytotoxic tolerance to 5-FU treatment in COAD.

### R273H and R248W Mutants in p53 Induce Chemoresistance by Upregulation of the CD44 Expression

**FIGURE 4:**
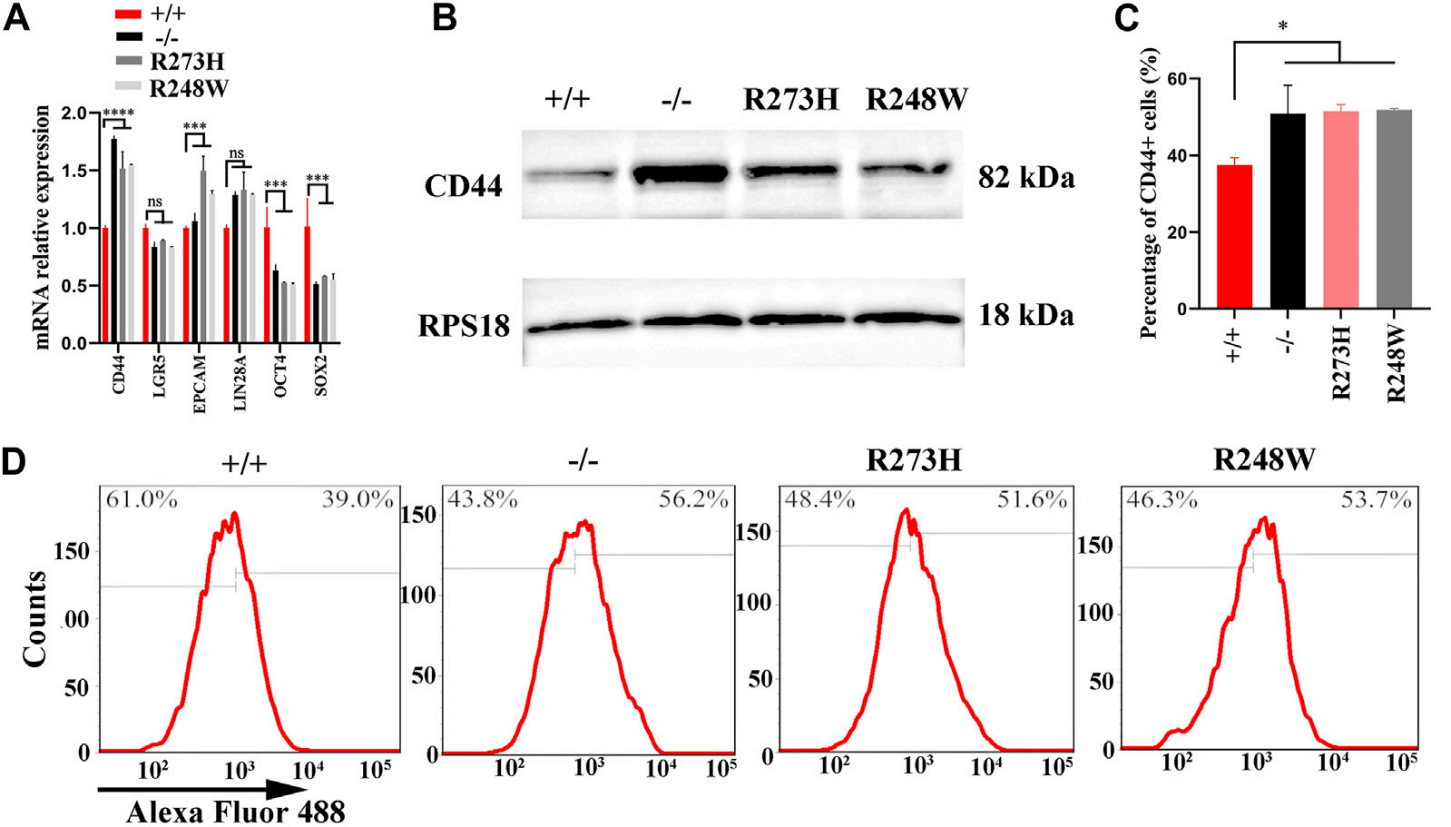
Expression levels of CD44 in different HCT116 cell lines. **(A,B)** mRNA and protein levels of CD44 in HCT116 p53 (+/+), p53 (-/-), p53 (R273H), and p53 (R248W). RPS18 was internal control. **(C,D)**. Images for CD44-positive cells in HCT116-derived cell lines. *p < 0.05. The error bar was from three independent experiments.

To understand how R273H and R248W of p53 exert their functions in 5-FU-resistance, we screened stemness-related hub genes at mRNA and protein levels. The Western blot results showed NANOG, SOX-2, and OCT4 expression levels were only slightly changed among the four HCT116 cell lines with different p53 statuses (Supplementary Figure S3). Considering the other stemness-related hub genes including CD44, LGR5, EPCAM, and LIN28 at the mRNA level in Figure 4A (*p* < 0.0001), the CD44 protein expression in Figure 4B, and the relationship between the deficient/mutant p53 and enhanced stemness phenotype of HCT116 cell lines, we chose CD44 as our potential target which contributes to the p53-mediated enhanced stemness phenotype in HCT116. In addition, the percentage of CD44 positive cells was increased in HCT116 cells with the deficient/mutant p53 status through flow cytometry, which was in line with the results of qPCR and Western blot (*p* < 0.05, Figures 4C,D). Simultaneously, CD44 was overexpressed in SW480 cells with mutp53 (Supplementary Figure S1C).

**FIGURE 5:**
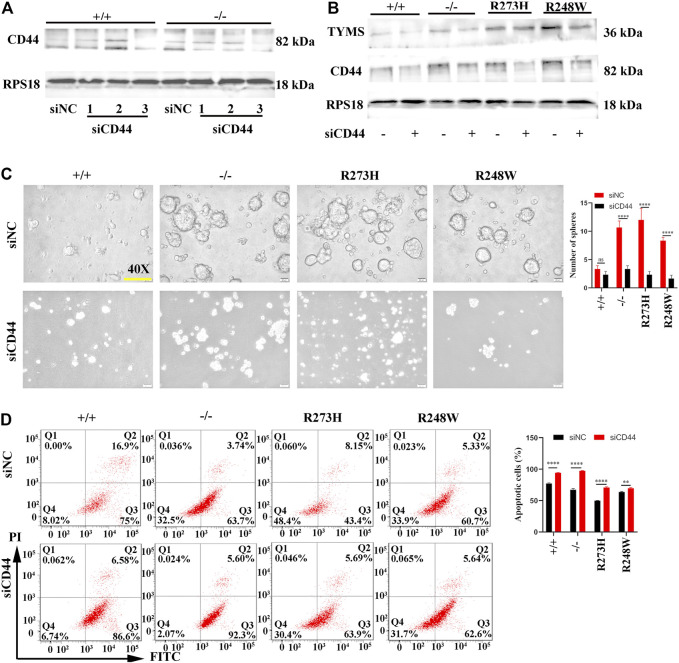
CD44 knockdown enhanced chemosensitivity in different HCT116 cell lines. **(A)** Western blot detection for the knockdown efficiency of three siCD44s in HCT116 p53 (+/+) and HCT116 53 (-/-) cells. siNC serves as the negative control. RPS18 was used as internal control of Western blot. **(B)** Protein levels of TYMS and CD44 in different HCT116 cell lines after knockdown of CD44 by siRNA. **(C)** Images of tumorsphere formation in CD44 knockdown HCT116 cell lines (magnification 400×). **(D)** Representative images for FITC-Annexin V/PI flow cytometry in siCD44-transfected HCT116 cell lines. The cells in Q2 and Q3 are considered as apoptotic cells. The error bar was from three independent experiments. (ns, no significance; **p < 0.01, ****p < 0.0001).

To further clarify whether the activated CD44 in mutated p53 HCT116 cell lines was responsible for chemoresistance, we knocked down the expression of CD44 by transfection of siRNAs specifically targeted to CD44 in R273H and R248W of p53 HCT116 tumor cells to examine the changes involved in 5-FU tolerance. After screening the knockdown efficiency of siCD44, we chose the third siRNA-targeted CD44 for the following rescue assays according to the CD44 protein expression levels (Figure 5A). TYMS was down-expressed in HCT116 p53 (-/-), p53 (R273H), and p53 (R248W) (Figure 5B). Tumor sphere formation rates were observed to be decreased in loss-of-function and mutated p53 HCT116 cell lines (*p* < 0.0001, Figure 5C). Flow cytometry further confirmed the apoptotic cells were increased when CD44 was interfered (*p* < 0.01, Figure 5D). Taken together, these data indicated that CD44 downregulation could rescue the chemoresistance in HCT116.

### Mutant p53 Promotes Colon Cancer Xenograft Growth and Chemoresistance *In vivo*

**FIGURE 6:**
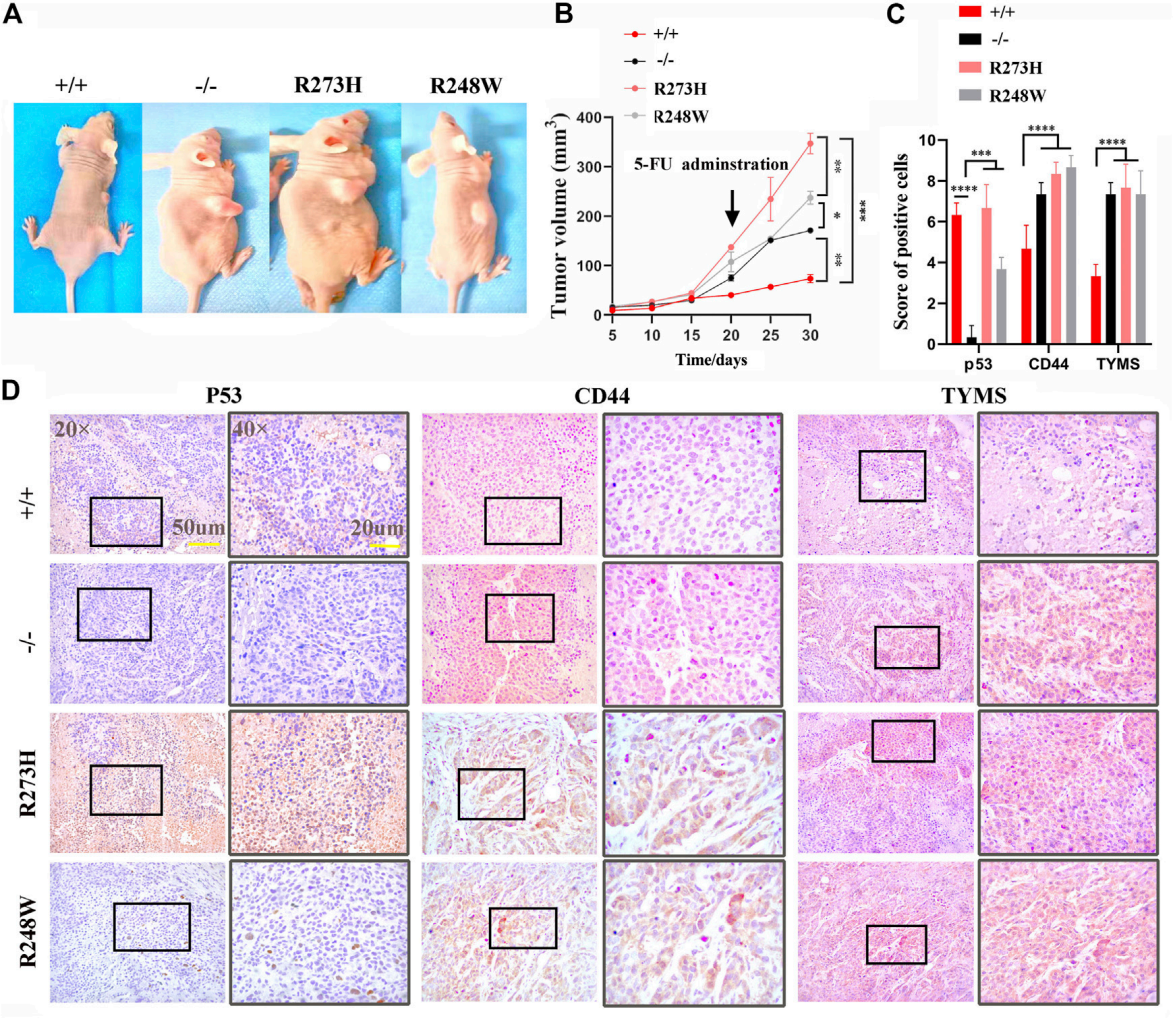
*In vivo* chemoresistance of deficient and mutant p53 HCT116 cell lines. **(A)** Tumor size of different HCT116-derived cell lines at 10 days post-transplantation in the right back of the mice. **(B)** Tumor growth curve of different HCT116-derived cell lines under 5-FU administration. **(C)** Immunohistochemistry scores of p53, CD44, and TYMS expressions in tumors. **(D)** Representative images for IHC staining of p53, CD44, and TYMS in tumor species. Brown areas show positive tumor cells. p53 almost located in nucleus, CD44, and TYMS are expressed in cytoplasm. *p < 0.05, **p < 0.01, ***p < 0.001, and ****p < 0.0001.

We established xenografts implanted into immunocompromised mice, dividing into four subgroups including HCT116 p53 (+/+), HCT116 p53 (-/-), HCT116 p53 (R273H), and HCT116 p53 (R248W). At 15 days after tumor xenograft transplantation, groups of HCT116 p53 (-/-), HCT116 p53 (R273H), and HCT116 p53 (R248W) exhibited greater tumors than HCT116 p53 (+/+) ones (Figure 6A). Identical to *in vitro* data, knockout of p53 and mutant p53 xenografts were dramatically resistant to 5-FU administration (*p* < 0.01, Figure 6B). Immunohistochemistry of p53, CD44, and TYMS was used to further verify that CD44 and TYMS showed intensively positive in HCT116 p53 (-/-), HCT116 p53 (R273H), and HCT116 p53 (R248W) groups, negatively related to the wild-type p53 (Figure 6C,D, *p* < 0.01). Collectively, *in vivo* data further confirmed that mutant p53 not only functioned as loss-of-function of deficient p53 but also favored tumor cells with more aggressive characteristics.

### R273H and R248W Mutants in p53 Induce Chemoresistance *via* the TCF21/CD44 Axis

**FIGURE 7:**
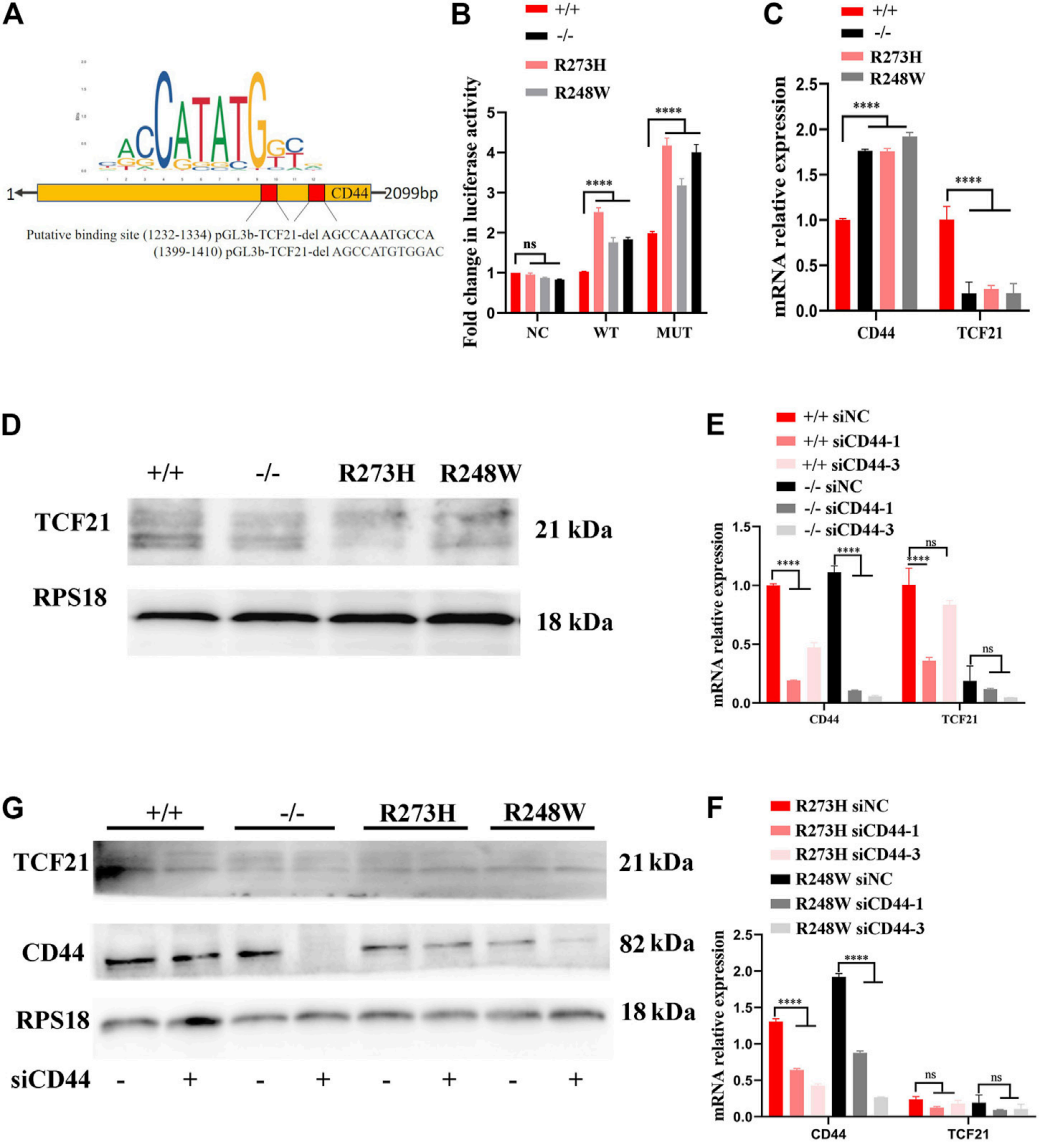
p53/TCF21/CD44 axis promotes chemoresistance in deficient and mutant p53 HCT116-derived cell lines. **(A)** Diagram for the luciferase reporter with the CD44 promoter, and two putative binding sites of TCF21 were deleted when the pGL3B-CD44mut plasmid was constructed. **(B)** Fold changes in luciferase activities of pGL3B-CD44wt with TCF21 and pGL3B-CD44mut without TCF21 binding sites in each HCT116-derived cell lines. **(C,D)** mRNA and protein levels in different HCT116 cells. RPS18 was performed as internal control. **(E–G)**. The expression changes at mRNA and protein levels in siCD44-transfected HCT116-derived cell lines. The error bar was from three independent experiments. (ns, no significance; ****p < 0.0001).

To unravel the underlying mechanism involved in HCT116 with p53 R273H and R248W mutations that sustained the overexpression of CD44, we analyzed the transcription motif in the CD44 promoter through JASPAR (https://jaspar.genereg.net/) to determine whether p53 directly regulated the CD44 expression. However, there was no efficient direct binding site of p53 in the CD44 promoter. Therefore, we searched for the possible transcription factors which could bind to the CD44 promoter and regulate the CD44 expression. We finally confirmed two putative binding sites of TCF21 as a candidate transcription factor of CD44. Wild-type and the two putative binding sites of TCF21 deletion sequences were cloned into the pGL3B vector, and luciferase assay was performed (Figure 7A). The result showed that the value of luciferase was strikingly increased when TCF21 was deleted, especially in the cases of deficient and mutant p53 (*p* < 0.0001, Figure 7B), suggesting that TCF21 could negatively regulate the CD44 activation. Additionally, the expression of TCF21 in tumor cells with different p53 statuses was confirmed by qPCR and Western blot. TCF21 was downregulated in deficient and mutant p53 HCT116 and SW480 cells both at mRNA and protein levels (Figures 7C,D; Supplementary Figure S1C). Moreover, knockdown of CD44 in HCT116 exerted no effect on the expression of TCF21, revealing the CD44 binding site upstream of TCF21-expressing cassettes by qPCR and Western blot (Figures 7E–G).

### Overexpression of TCF21 Rescues Dysfunctions of p53

**FIGURE 8:**
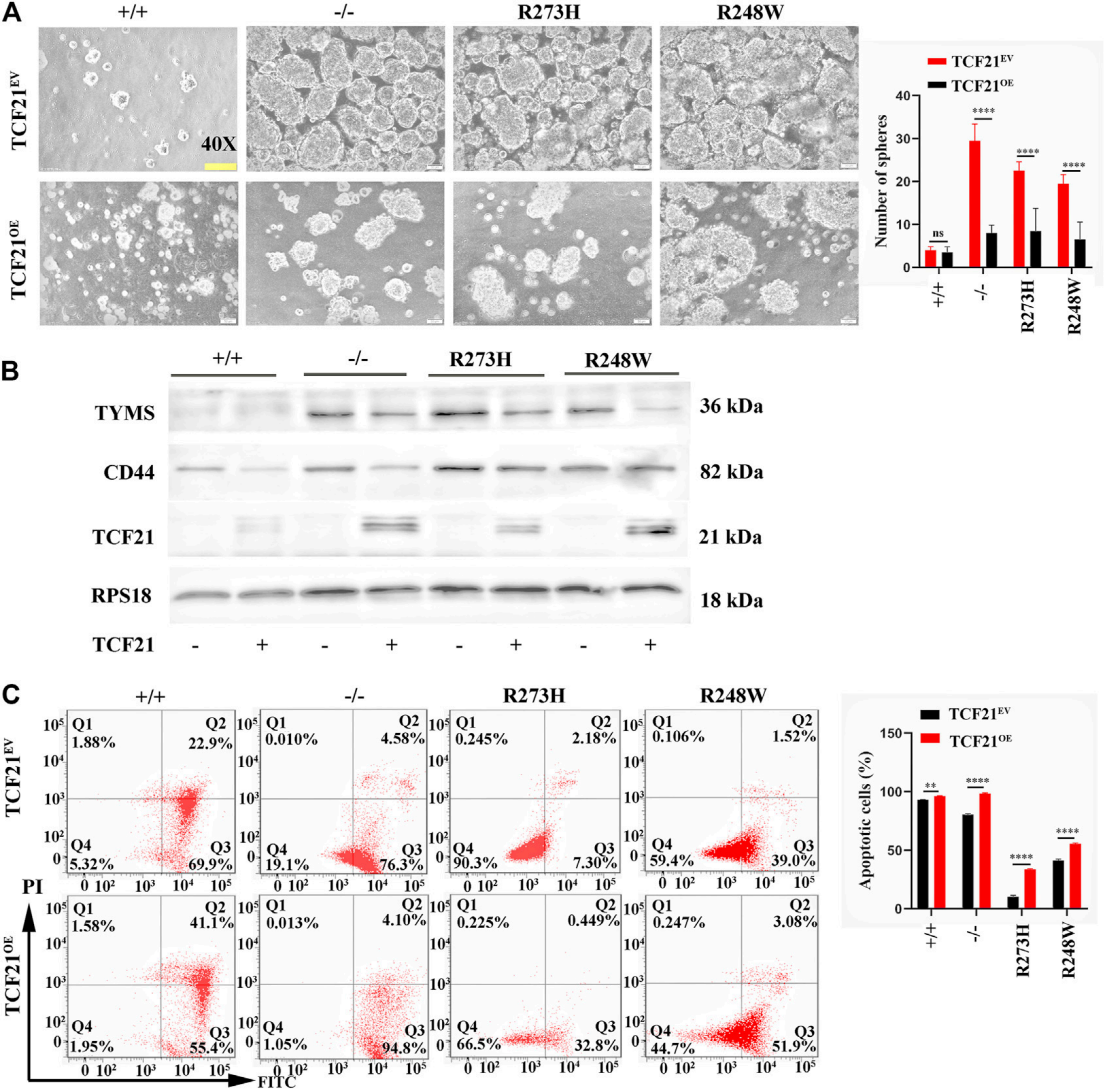
TCF21 overexpression rescues chemoresistance in deficient and mutant p53 HCT116 cell lines. **(A)** Images of tumorsphere formation in TCF21 overexpressed HCT116-derived cell lines (magnification ×400). **(B)** Protein levels of TYMS, CD44, and TCF21 in different HCT116-derived cell lines. RPS18 was internal control. **(C)**. Representative images for FITC-Annexin V/PI flow cytometry in TCF21 overexpressed HCT116-derived cell lines. The cells in Q2 and Q3 are considered as apoptotic cells. The error bar was from three independent experiments (ns, no significance; **p < 0.01, ****p < 0.0001).

To examine whether the inactivation of TCF21 in mutated cancer cells was responsible for CD44-induced 5-FU resistance, we elevated the expression of TCF21 in knockout and mutant p53 tumor HCT116–derived cell lines to verify the effects on chemoresistance. Sphere formation rates were observed to be obviously decreased in TCF21-activated HCT116 p53 (-/-), p53 (R273H), and p53 (R248W) cell lines (*p* < 0.0001, Figure 8A), but mere changes in the cells harbor wild-type p53. Western blot also showed that TYMS and CD44 were decreased in the TCF21 overexpression HCT116 cell lines (Figure 8B). Furthermore, flow cytometry confirmed the apoptotic cells were increased when TCF21 was elevated (*p* < 0.01, Figure 8C). Together, the results further provide evidence that mutant p53 could regulate chemoresistance of colorectal cancer through the TCF21/CD44 axis; expression changes of either TCF21 or CD44 could rescue the mutant p53-induced chemoresistance in colorectal carcinoma.

## Discussion

p53 mutation is one of the crucial attempts to develop survival ways for resisting radiotherapy and chemotherapy. Although several studies have identified a portion of genes regulated by mutant p53, targeting anyone of the downstream of the mutant p53 pathway seems too hard to completely restore the chemosensitivity. Furthermore, therapeutic strategies by targeting mutant p53 are still far from clinical application, though it is the best druggable target theoretically. In this scenario, there is a pressing need to identify more effective and convenient targets to overcome mutant p53-driven chemoresistance.

Here, we first find oncogenic properties of enhanced stemness, metastasis, and chemoresistance conferred by deficient or mutant R273H and R248W of p53 in HCT116 cell lines. Consistent with extracorporal results, mice harbored HCT116 p53 (-/-) and the two mutant HCT116-derived cell lines develop larger tumors than those with the wild-type TP53. Importantly, these tumors with deficient or mutant p53 resisted to 5-FU administration, with high levels of CD44 and TYMS. These malignant characteristics endowed by mutant p53 are considered to contribute to poor survival in human cancers and also give insights into our findings in TCGA database. We recognized that the p53 mutation was significantly implicated in the disease-free interval of colorectal carcinoma. The patients with tumor recurrence harbored a high mutant frequency of p53 than those free for tumors. Both mTOR and VEGF play a crucial role in tumor metastasis and are upregulated in the mutant p53 group. Moreover, genes from the cohort with mutant p53 enriched in tumor-associated pathways such as cell cycle and DNA transcription. Compared with the wild-type p53 group, the genes involved in cell adhesion, and the p53 signaling pathway and cell cytotoxicity are also remarkably downregulated in antitumor pathways.

Here, we found that the major aggressive properties which mutant and deficient p53 endow cancer cells with elevated invasion, metastasis, and resistance to 5-FU chemotherapeutic treatment are also integral to cancer stem cells. Considering the fold changes of the cancer stem cell biomarkers, we chose CD44 as our potential target. Fortunately, our further results directly supported the conclusion that gain-of-function of mutant p53 yielded the impaired cytotoxicity of 5-FU through an increase in CD44-induced stemness in colorectal cancer.

The new finding pushes us to investigate the underlying mechanism by which deficient and mutant p53 induce colorectal cancer chemoresistance, which might unravel new targets for improving the prognosis of patients with deficient and mutant p53. Accordingly, we performed studies *in vitro* and found that CD44 was significantly upregulated in mutant p53 using homologous R273H and R248W of p53 in HCT116-derived cell lines. The result was in line with the conclusion of Solomon et al. They found that colorectal cancer cells with mutant p53 harbor an increased population of CD44-, LGR5-, and ALDH-positive cells (Solomon et al., 2018). Several crucial pathways, which both promote chemotherapeutic resistance and are also central to the cancer stem cells, have been explored. For instance, ABC transporters abundantly expressed in cancer stem cells, and pump drugs out of the cancer cells. MDR1, one of the ABC family members, remains a low level in normal cells with wild-type p53 and yet holds a high level in mutant p53 cancer cells (Chin et al., 1992). Simultaneously, cancer stem cells and mutant p53 cancer cells shared the common anti-apoptotic signature as the Bcl2 family (Wang et al., 2015; Zhou et al., 2019). Despite them having the relationship between cancer stem cells and mutant p53 cancer cells, the mechanism regarding how deficient and mutant p53 induce drug tolerance and the role of cancer stem cells in the process of deficient or mutant p53 induced drug tolerance was far from elucidated.

Promisingly, our new finding demonstrated that CD44 could exert a direct role in increasing the TYMS expression. TYMS encodes thymidylate synthase, which is the primary target of 5-FU, impairs the drug sensitivity in colorectal carcinoma, and is found to activate aggressive cancer cells (Zhou et al., 2012; Abdallah et al., 2015). In our study, TYMS was increased in knockout and mutant p53 groups both *in vitro* and *in vivo*. Knockdown of CD44 in HCT116 cell lines decreases the TYMS expression, which indicates that HCT116 cells with deficient p53 or R273H and R248W mutant p53 are more resistant to 5-FU. Moreover, attenuated sphere formation and anti-apoptosis abilities were observed in CD44-silenced HCT116 cell lines, which further confirm deficient or mutant p53-driven stemness and chemoresistance to 5-FU through upregulation of CD44. Given that CD44 is a biomarker of the cancer stem cell biomarker, collectively, our data indicate that we may build a bridge between mutant p53-driven chemoresistance and the CD44-enhanced stem-like phenotype in colon cancers.

Many efforts have been made to elucidate the impact of p53 mutations on cancer stemness. One scenario describes that mutant p53 contributes to somatic cell reprogramming towards pluripotent and immature (Sarig et al., 2010). Another scenario proposes that mutant p53 induces a series of signaling pathways that support the cell survival and maintenance of cancer stem cells (Escoll et al., 2017). The third scenario emphasizes the function of mutant p53 on cancer stem cell energetic metabolism, especially the mevalonate pathway (Sorrentino et al., 2014). However, the molecular mechanism of deficient or mutant p53-driven stemness still remains largely unexplored.

In our study, TCF21, a transcription factor that remained activated in normal cells, harbors wild-type p53 and is suppressed in loss-of-function and mutant p53 HCT116 cancer cells. Moreover, overexpression of TCF21 could overthrow the stemness and chemoresistance in HCT116-derived cell lines. First, TCF21 has been revealed in uterine corpus endometrial carcinoma cases according to the microarray studies in 2012 (Chiyoda et al., 2012; Gardi et al., 2014). It is considered as a tumor suppressor in controlling cell growth, migration, and apoptosis and is observed to be abundantly expressed in cells with wild-type p53 (Hu et al., 2014; Wang and Scadden, 2015). More recently, Li et al. demonstrated that TCF21 functions as an antitumor factor *via* the MEK/ERK signaling pathway (Li et al., 2018).

**FIGURE 9:**
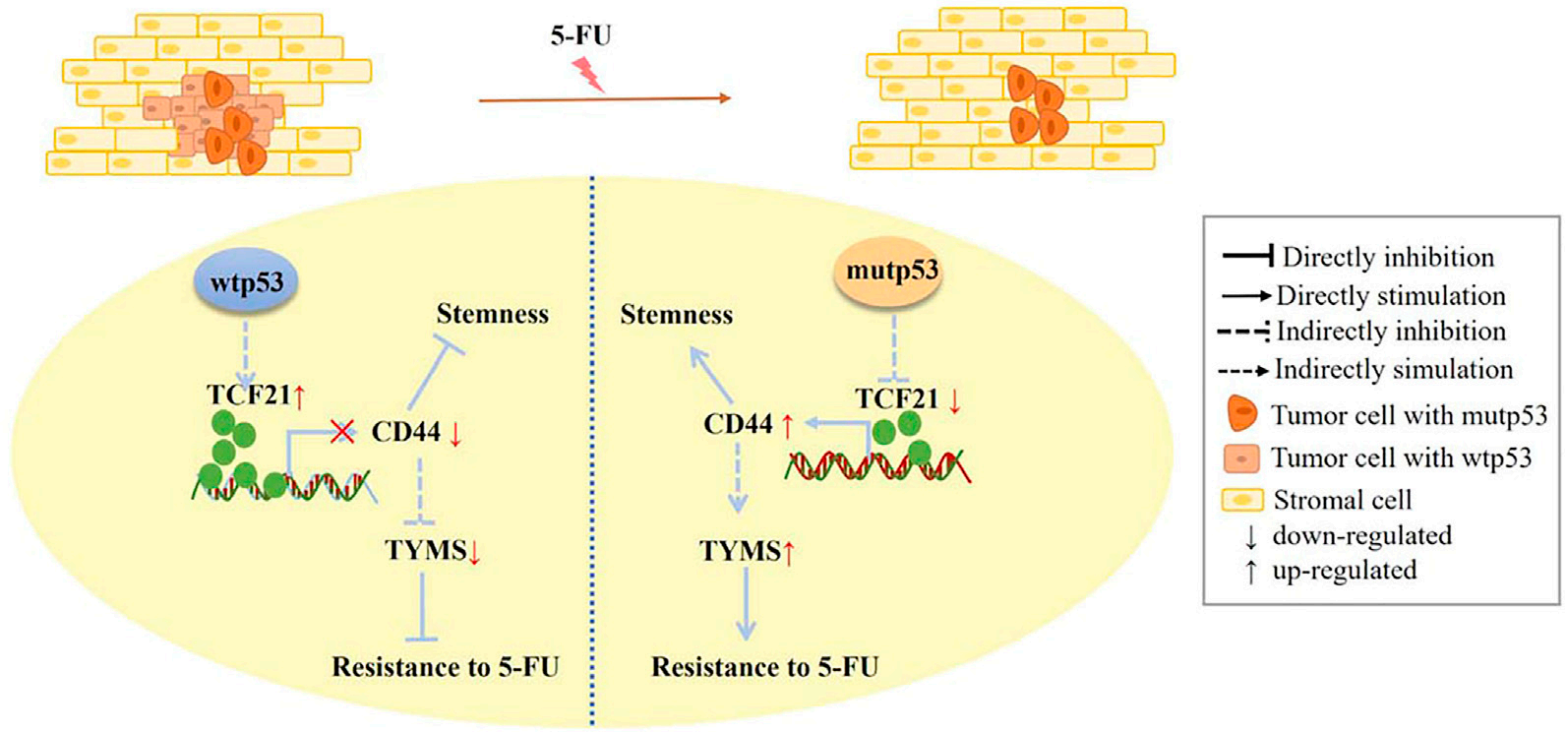
Scheme of the novel mechanism by which p53 regulates chemoresistance to 5-FU *via* TCF21/CD44 axis-mediated enhanced stemness in colorectal carcinoma. TCF21 is rich in tumor cells with wtp53 and can directly bind to the CD44 promoter to suppress the expression levels of CD44, leading to the decrease of TYMS. In contrast, CD44 is abundant in tumor cells with mutp53 because of a low level of TCF21, resulting in the increase of TYMS, thus enhancing chemoresistance, stemness, and proliferation of colorectal carcinoma cells.

We demonstrated that TCF21 could directly inactivate CD44 by binding to the CD44 promoter in HCT116 cells with wild-type p53. On the contrary, deletion of TCF21 strikingly increases the CD44 expression in knockout and mutant p53 (R273H and R248W) colorectal cancer cells. Importantly, rescue of the expression level of TCF21 could suppress the CD44 expression. Taken together, we proposed a potential novel mechanism by which mutant p53 triggered chemoresistance *via* TCF21/CD44 axis-mediated enhanced stemness (Figure 9).

Though the mechanism of mutant p53-dependent TCF21 activation has not yet been explored in this study, we found that p53 mutants in Arg273 and Arg248 changed the structure of wild-type p53 from linear to circular online (http://missense3d.bc.ic.ac.uk/∼missense3d/), suggesting a potential mechanism by which mutant p53 decreased the TCF21 expression *via* removing the original binding site for TCF21 (Supplementary Figure S4). Obviously, further evidence is needed in future to verify our postulate.

## Conclusion

In conclusion, our data, for the first time, not only provides direct evidence of mutant p53-induced chemoresistance by promoting cancer stemness in colorectal carcinoma but also highlights the potential targets of TCF21/CD44 against mutant p53-driven drug resistance.

## Data Availability

The datasets presented in this study can be found in online repositories. The names of the repository/repositories and accession number(s) can be found in the article/Supplementary Material.
