# Correction: Deficient or R273H and R248W mutations of p53 promote chemoresistance to 5-FU via TCF21/CD44 axis-mediated enhanced stemness in colorectal carcinoma

**DOI:** 10.3389/fcell.2026.1857068

**Published:** 2026-05-27

**Authors:** Xiaolei Gao, Xuan Zheng, Yixin Zhang, Liying Dong, Liangjie Sun, Na Zhao, Chong Ding, Zeyun Ma, Yixiang Wang

**Affiliations:** 1 Central Laboratory, Beijing, China; 2 Department of Oral and Maxillofacial Surgery, Beijing, China; 3 National Engineering Laboratory for Digital and Material Technology of Stomatology, Beijing, China; 4 Beijing Key Laboratory of Digital Stomatology, Beijing, China; 5 Department of Restorative Dentistry and Biomaterials Sciences, Harvard School of Dental Medicine, Boston, MA, United States; 6 Shanghai Stomatological Hospital, Fudan University, Shanghai, China; 7 Department of VIP Service, Peking University School and Hospital of Stomatology, Beijing, China

**Keywords:** colorectal carcinoma, deficient or mutant p53, R273H, R248W, stemness, TCF21/CD44 axis, chemoresistance

There was a mistake in Figure 3 as published. The flow cytometry data in Figure 3B were misused, which occurred unintentionally during the figure preparation process. The corrected Figure 3 appears below.

**FIGURE 3:**
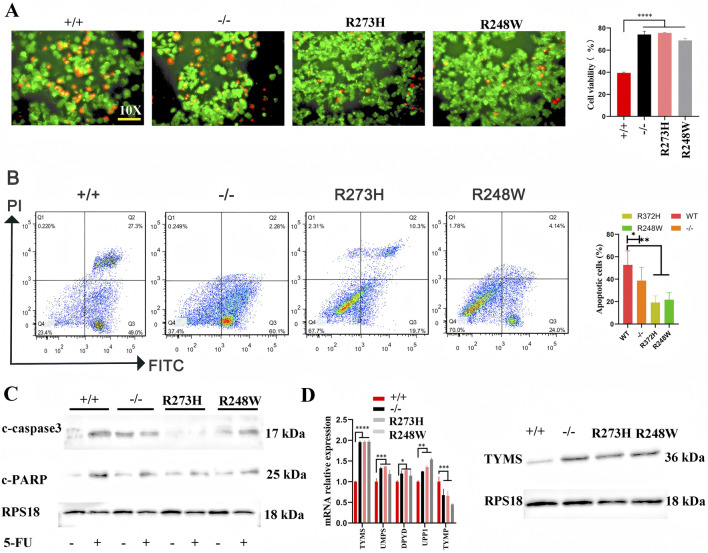
Anti-apoptosis and 5-FU resistance in HCT116 with different p53 statuses. **(A)** Representative images for live-dead staining in HCT116-derived cell lines. Red dot represents dead cells, and green dot shows alive cells. **(B)** Representative images for FITC-Annexin V/PI flow cytometry in HCT116-derived cell lines. The areas of Q2 and Q3 are considered as apoptotic cells. **(C)** The expressions of cleaved-caspase3 and cleaved PARP protein in HCT116-derived cell lines. **(D)** mRNA and protein levels of TYMS in different HCT116-derived cell lines. ****p < 0.0001. The error bar was from three independent experiments.

The ethics statement was erroneously given as “The animal study was reviewed and approved by The Institutional Animal Care and Use Committee at Peking University Health Center”. The correct ethics statement is “The animal experiments were approved by the Institutional Animal Care and Use Committee at Peking University Health Center (LA 2019319)”.

The original article has been updated.

