# Supplementary figures and images for: Deficient or R273H and R248W Mutations of p53 Promote Chemoresistance to 5-FU via TCF21/CD44 Axis-Mediated Enhanced Stemness in Colorectal Carcinoma
Source: Front Cell Dev Biol. 2022 Jan 5;9:788331. doi: 10.3389/fcell.2021.788331 (PMC8766496; doi:10.3389/fcell.2021.788331)

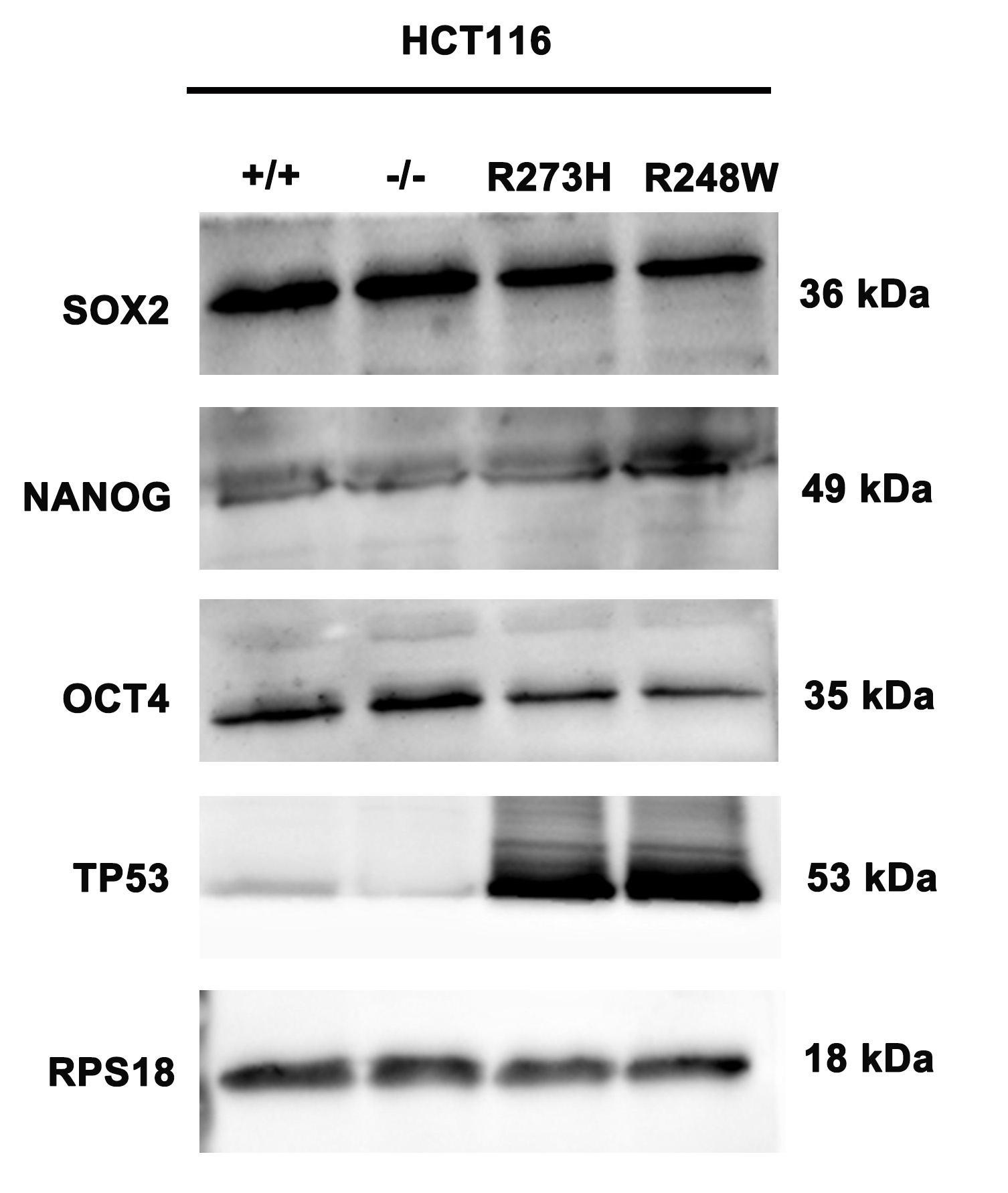

Supplement: Supplementary file 1 [file Image3.JPEG]

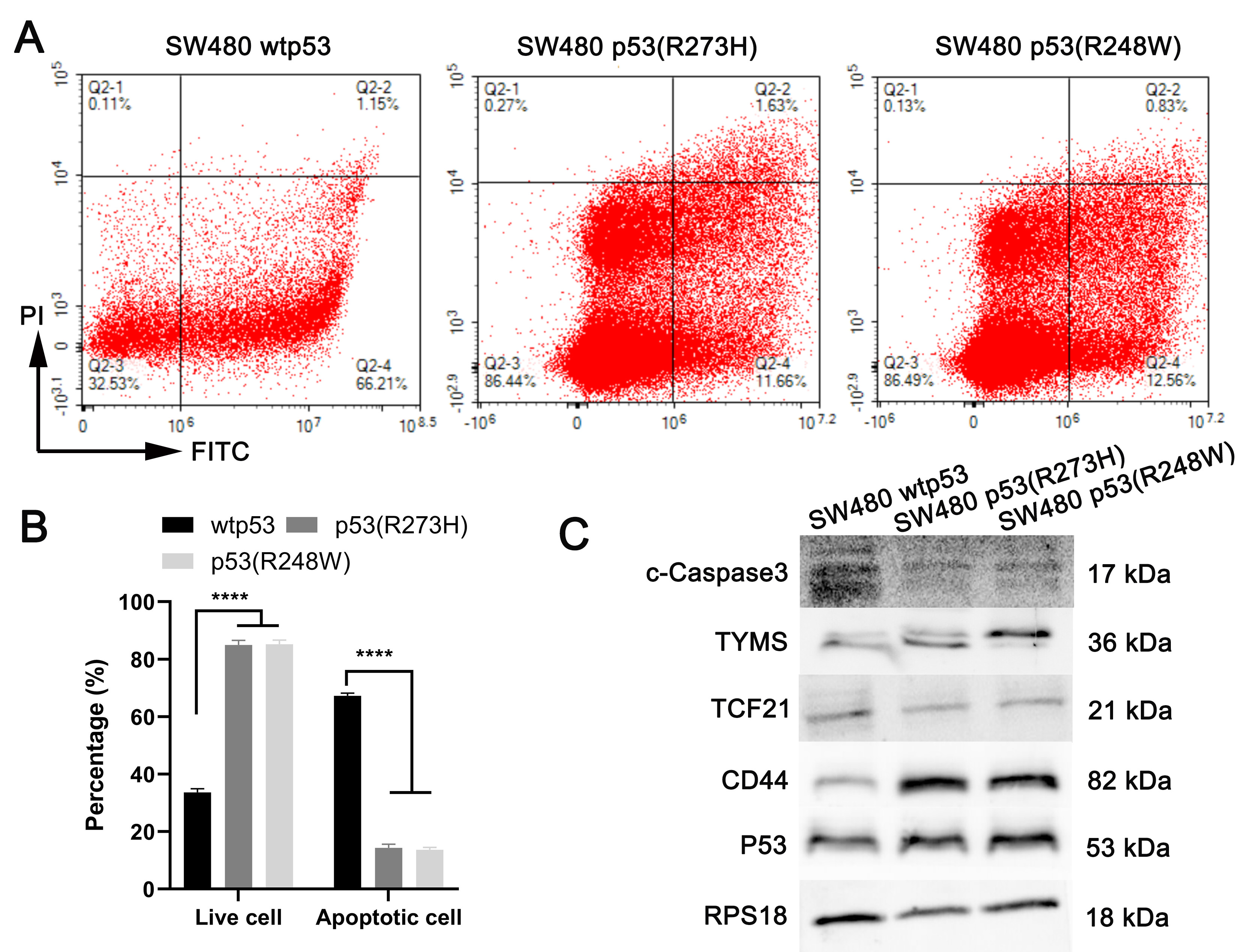

Supplement: Supplementary file 2 [file Image1.JPEG]

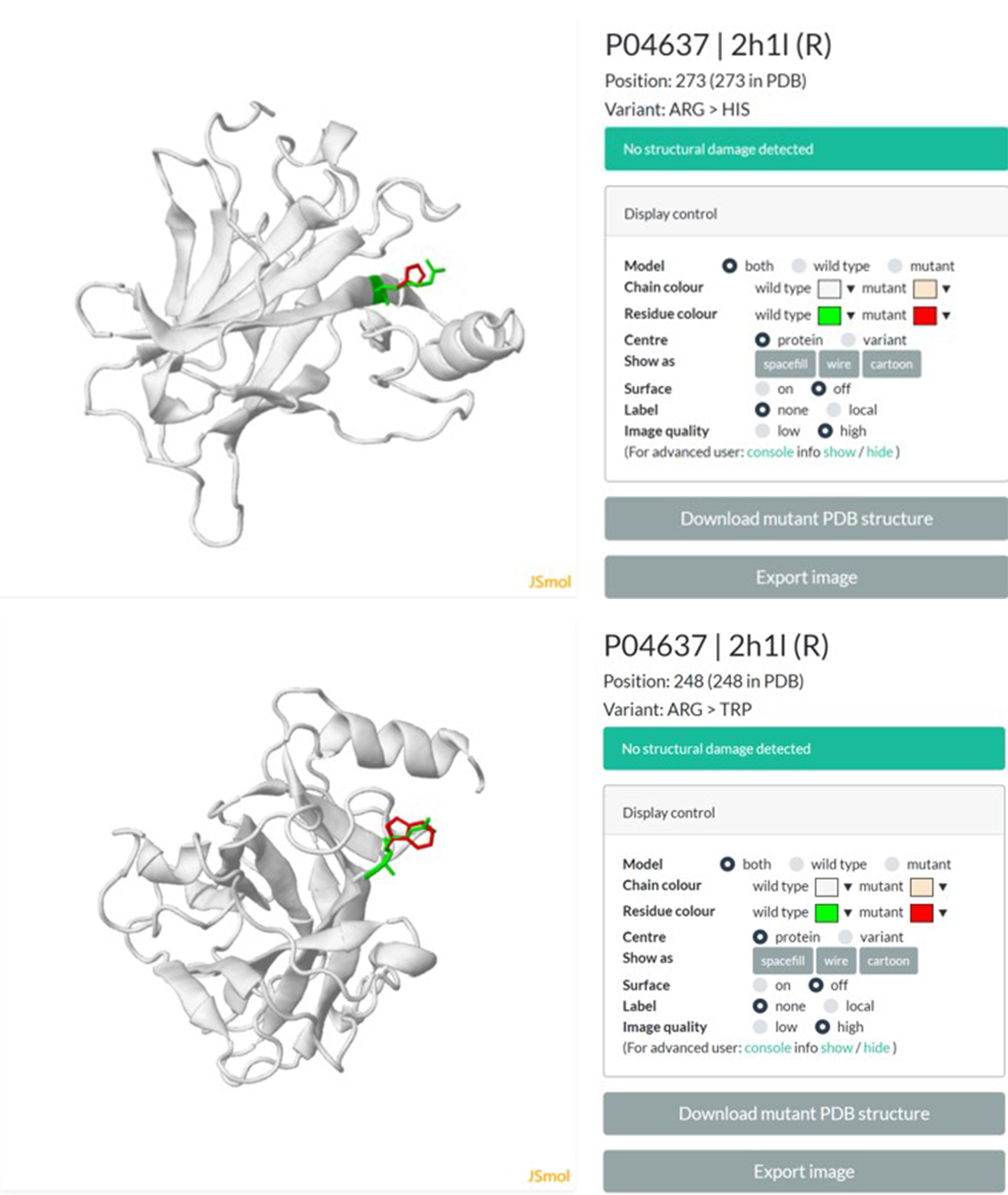

Supplement: Supplementary file 3 [file Image4.JPEG]

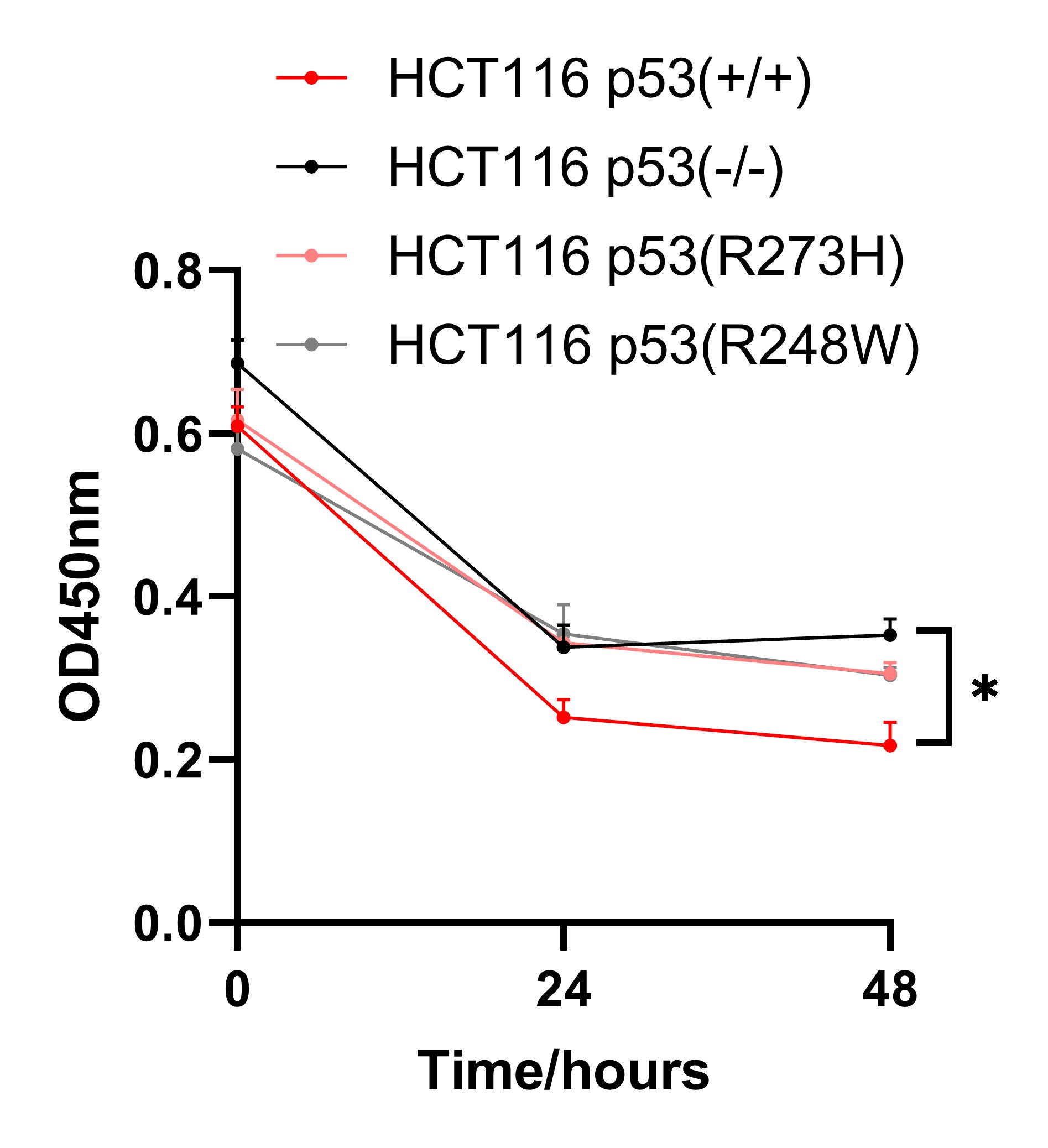

Supplement: Supplementary file 4 [file Image2.JPEG]
